# Vaccine-derived poliovirus surveillance in China during 2001–2013: the potential challenge for maintaining polio free status

**DOI:** 10.1186/s12879-017-2849-z

**Published:** 2017-12-02

**Authors:** Hai-Bo Wang, Hui-Ming Luo, Li Li, Chun-Xiang Fan, Li-Xin Hao, Chao Ma, Qi-Ru Su, Hong Yang, Kathleen H. Reilly, Hua-Qing Wang, Ning Wen

**Affiliations:** 1 Chinese Center for Disease Control and Prevention, 27 Nanwei Road, Beijing, 100050 People’s Republic of China; 2 Peking University Clinical Research Institute, Xueyuan Rd 38#, Haidian District, Beijing, 100191 People’s Republic of China; 3 Independent Consultant, New York City, NY USA

**Keywords:** Vaccine derived poliovirus, Importation, China, Polio eradication, Wild poliovirus

## Abstract

**Background:**

The goal of polio eradication is to complete elimination and containment of all wild, vaccine-related and Sabin polioviruses. Vaccine-derived poliovirus (VDPV) surveillance in China from 2001–2013 is summarized in this report, which has important implications for the global polio eradication initiative.

**Methods:**

Acute flaccid paralysis (AFP) cases and their contacts with VDPVs isolated from fecal specimens were identified in our AFP surveillance system or by field investigation. Epidemiological and laboratory information for these children were analyzed and the reasons for the VDPV outbreak was explored.

**Results:**

VDPVs were isolated from a total of 49 children in more than two-thirds of Chinese provinces from 2001–2013, including 15 VDPV cases, 15 non-polio AFP cases and 19 contacts of AFP cases or healthy subjects. A total of 3 circulating VDPVs (cVDPVs) outbreaks were reported in China, resulting in 6 cVDPVs cases who had not been vaccinated with oral attenuated poliomyelitis vaccine. Among the 4 immunodeficiency-associated VDPVs (iVDPVs) cases, the longest duration of virus excretion was about 20 months. In addition, one imported VDPV case from Myanmar was detected in Yunnan Province.

**Conclusions:**

Until all wild, vaccine-related and Sabin polioviruses are eradicated in the world, high quality routine immunization and sensitive AFP surveillance should be maintained, focusing efforts on underserved populations in high risk areas.

## Background

Polio eradication was declared as a programmatic emergency for global public health by the World Health Organization (WHO) [1]. Major progress has been made since the Global Polio Eradication Initiative (GPEI) was launched by the World Health Assembly in 1998. GPEI has reduced the number of wild polioviruses (WPV) cases by >99%, from an estimated 350,000 in 1988 to 37 reported cases in 2016 [2]. Eradication of type 2 WPV was certified in 2015, [3] and type 3 WPV was last detected in November 2012 in Nigeria [4]. However, indigenous transmission of WPV continues in the remaining three countries (Afghanistan, Nigeriaand Pakistan) [2].

WPV had been historically endemic in China, with approximately 20,000 paralytic cases reported annually since the early 1950s [5]. Based on high routine vaccination coverage, sensitive acute flaccid paralysis (AFP) surveillance, high quality National Immunization Days and supplementary immunization activities (SIAs), the number of poliomyelitis declined dramatically. Eventually, the last polio case infected by indigenous WPV was reported in September 1994 in China, and the Western Pacific Region, which encompasses China, has been certified as being polio free since October 2000 [5]. As it shares border with endemic countries, China has experienced WPV importation for many times before being polio-free: 1995 and 1996 in Yunnan Province [6], 1999 in Qinghai Province [7]. In August 2011, after maintaining polio-free for more than 10 years, a WPV outbreak occurred in Xinjiang Uyghur Autonomous Region, China following importation from Pakistan [8, 9].

Oral attenuated poliomyelitis vaccine (OPV), which is the primary tool for the global eradication of WPV, possess its own significant advantages including easy administration by mouth, inducing both humoral and intestinal immunity and relatively low cost [10, 11]. However, due to a high liability of genetic mutation and recombination with other enteroviruses [12], one disadvantage associated with OPV is the rare occurrence of vaccine-associated paralytic poliomyelitis (VAPP) and emergence of genetically divergent vaccine-derived polioviruses (VDPVs) [13, 14]. The outbreaks of circulating VDPVs (cVDPVs) in Nigeria [15], have indicated the potential risk of prolonged transmission of the vaccine virus among populations with non-optimal immunity.

The goal of polio eradication is to complete elimination and containment of all wild, vaccine-related and Sabin polioviruses [16]. For eradicating type 2 polioviruses in the world, the transition from trivalent OPV to bivalent OPV was implemented in April 2016 [4]. Therefore, developing a post-eradication vaccination strategy to minimize the risk of VDPVs is urgently needed, but is also confronted with many challenges, including risk assessment of VDPV, vaccine funding, clearing long-term shedding of vaccine virus by immune-deficient person, and OPV stockpiles [16]. Therefore, characterization of VDPV may provide additional and valuable information for making vaccination policies [17]. VDPV surveillance is summarized in this report which describes VDPV detected between 2001 and 2013 in China, as well as the results of epidemiological and laboratory investigations. These data provide meaningful insights for polio eradication as China has the largest population to vaccinate after being polio free for more than 10 years.

## Methods

### AFP cases surveillance in China

AFP case surveillance is based on passive reporting by hospitals and active surveillance by the Center for Disease Control and Prevention (CDC) at the county level. Hospitals at the county level or above and some township hospitals in areas with dense populations or bordering with endemic countries are included in AFP surveillance system. AFP cases were reported to the county CDC through telephone or written report by hospital physicians prior to 2012, and AFP cases have been directly reported into a real-time online AFP surveillance system since 2012. After receiving reports of AFP cases, county CDC staff will routinely conduct epidemiological investigations, collect stool specimens, and assess residual paralysis 60 days after the onset of paralysis [9]. In addition, county CDC staff are responsible for reviewing medical records of all hospitals in the surveillance system every 10 days, regardless of whether AFP cases are reported.

Based on clinical information and stool specimen test results, AFP cases are classified by the provincial Polio Expert Committee (PEC) as follows: WPV cases, clinically compatible polio case, non-polio AFP cases, and VDPV cases.

### Isolation and characterization of poliovirus isolates

Stool specimens collected by the county CDC are subsequently forwarded to the provincial polio laboratory for poliovirus isolation. Viral isolation is performed on L20B and RD cell cultures, and viral isolates are primarily identified by micro-neutralization tests using poliovirus type-specific rabbit polyclonal antiserum (National Institute for Public Health and the Environment, Bilthoven, the Netherlands). Intratypic differentiation (ITD), VP1 sequencing and other necessary laboratory tests are conducted for determining poliovirus origin (wild or vaccine related) [18]. ITD is performed by polymerase chain reaction (PCR)–restriction fragment–length polymorphism (RFLP), real-time reverse transcription PCR and by enzyme-linked immunosorbent assay (ELISA). All tests are processed according to the standard guidelines recommended by WHO [19].

### Case ascertainment and definition

An AFP case is defined as any child younger than 15 years of age with acute onset of flaccid paralysis, or a person of any age with paralytic illness in whom poliomyelitis is suspected. It is required that at least 80% of AFP cases have adequate stool specimens collected (two stool specimens of sufficient quantity for laboratory analysis, collected at least 24 h apart, within 14 days after the onset of paralysis). A non-polio AFP case is defined as any AFP case who is tested negative for poliovirus on adequate stool specimens or who is judged by the provincial PEC to not be polio-compatible.

According to the National AFP cases surveillance guidelines, a VDPV case was defined as an AFP case from whom Sabin-related poliovirus (≥10 nucleotides (nt) VP1 substitutions for type 1, type 2 and type 3 before 2010, ≥10 nt VP1 substitutions [type 1 and type 3] or ≥6 nt VP1 substitutions [type 2] since 2010) was isolated from ≥1 stool specimen, but without WPV isolated, and who was also determined to be polio compatible by the provincial PEC. VDPVs are further categorized as 1) cVDPVs, when there is evidence of person-to-person transmission; 2) immunodeficiency-associated VDPVs (iVDPVs), which are isolated from persons with primary immunodeficiencies who have prolonged VDPV infections after exposure to OPV [20]; and 3) ambiguous VDPVs (aVDPVs), which are isolated from persons with no known immunodeficiency and no transmission evidence or a sewage sample whose ultimate source is not identified [21].

### Statistical analysis

Statistical tests were performed using SAS 9.3 software (SAS Institute Inc., Cary, NC, USA). Comparison between groups was done using chi-square and Fischer exact tests for categorical variables. Wilcoxon rank-sum analyses were used to compare age differences. A *p*-value of <0.05 was considered the cut-off for statistical significance for all analyses.

## Results

### Descriptive epidemiology

During 2001–2013, VDPVs were isolated from a total of 49 children, including 15 VDPV cases, 15 non-polio AFP cases and 19 contacts of AFP cases or healthy subjects (Table 1). Slightly more than half (51.0%, 25/49) children were male, with age ranging from 2 months to 13 years (median ± interquartile range (IQR), 1.4 ± 3.3 years). Seven (46.7%) VDPV cases were not immunized with OPV, and VDPV cases were less likely to receive OPV immunization (*P* < 0.01). Based on the first isolation virus, a total of 28 (57.1%) children had 10 or less nucleotide substitutions in the VP1 region, and only 5 (10.2%) children had more than 15 nucleotide substitutions; 15 (41.7%) and 16 (44.4%) children had ≤10 and 11–15 nucleotide substitutions in the VP1 region after excluding the type 2 VDPVs with <9 nucleotide substitutions. Among the 5 children with more than 15 nucleotide substitutions, one child was an iVDPV case, one non-polio AFP case, and three contacts. The 15 non-polio AFP cases were classified by the provincial PEC as hypokalemia (*n* = 4), Guillain-Barré syndrome (*n* = 3), transverse myelitis (*n* = 3), transient limb paralysis (*n* = 2), hand-foot-mouth disease (*n* = 2) and central nervous dysfunction (*n* = 1).

**Table 1:** Demographic characteristics, immunization history and laboratory testing results by the types of children (VDPV case, non-polio AFP case and healthy population) in China during 2001–2013

		VDPVs case	Non-polio AFP case	Healthy population
Sex	Male	11 (73.3)	7 (46.7)	7 (36.8)
	Female	4 (26.7)	8 (53.3)	12 (63.2)
Age (Median ± interquartile range)		1.1 ± 1.3	1.4 ± 1.8	3.9 ± 6.6
OPV immunization history	0 dose	7 (46.7)	2 (13.3)	3 (15.8)
	1–2 dose	3 (20.0)	1 (6.7)	2 (10.5)
	≥3 dose	5 (33.3)	10 (66.7)	4 (21.1)
	Unknown	0 (0.0)	2 (13.3)	10 (52.6)
Nucleotide substitution in VP1 region ^a^	≤10	7 (46.7)	12 (80.0)	9 (47.4)
	11–15	7 (46.7)	2 (13.3)	7 (36.8)
	≥ 16	1 (6.7)	1 (6.7)	3 (15.8)
Serotypes of VDPVs ^a^	Type 1	6 (40.0)	3 (20.0)	11 (57.9)
	Type 2	4 (26.7)	10 (66.7)	6 (31.6)
	Type 3	3 (20.0)	2 (13.3)	2 (10.5)
	Type 2 + 3	2 (13.3)	0 (0.0)	0 (0.0)

^a^Based on the first isolation virus

**Fig. 1:**
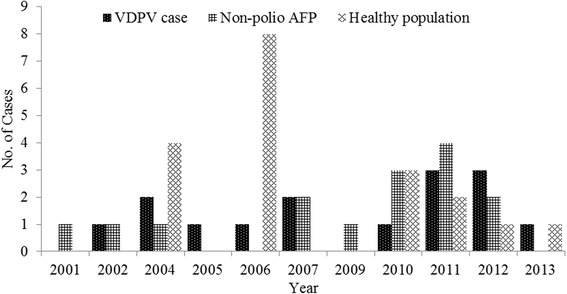
The number of children from whom VDPVs were isolated by year of identification and the types of children (VDPV case, non-polio AFP case and healthy population) in China during 2001–2013. VDPV, vaccine-derived polioviruses; AFP, acute flaccid paralysis; healthy population, contacts of AFP cases or healthy subjects

**Fig. 2:**
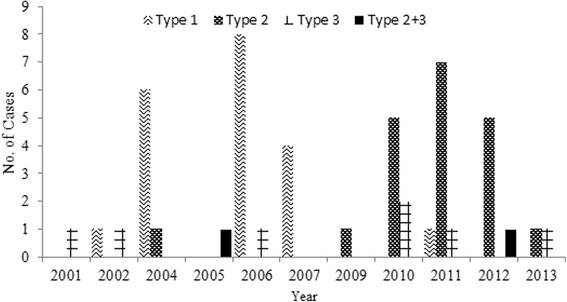
The number of children from whom VDPVs were isolated by year of identification and the types of serotypes of poliovirus in China during 2001–2013. VDPV, vaccine-derived polioviruses

**Fig. 3:**
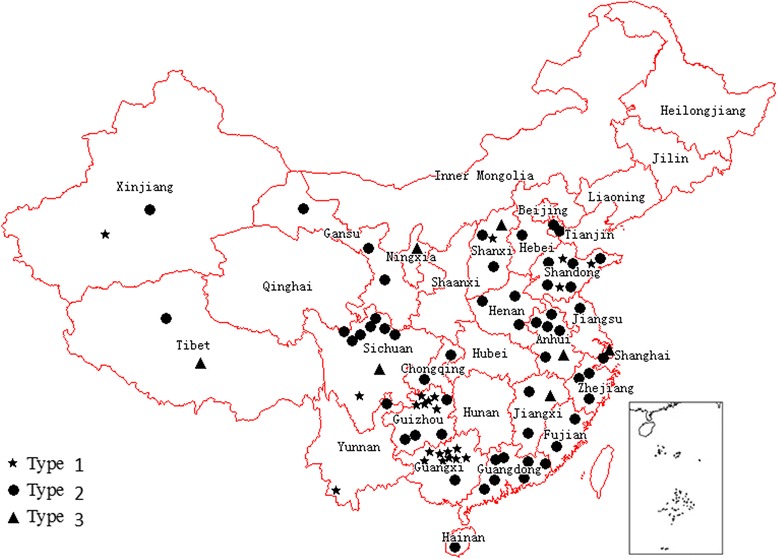
Geographical distribution of VDPVs for AFP cases or healthy population by types of serotypes of poliovirus in China during 2001–2013. VDPV, vaccine-derived polioviruses; AFP, acute flaccid paralysis; healthy population, contacts of AFP cases or healthy subjects. The placement of the symbols for the geographical location of the VDPVs is random within each province

Fifteen VDPV cases were further classified as cVDPV (*n* = 6), iVDPV (n = 4), and aVDPV (*n* = 5) cases. One VDPV case was detected in 2002, two cases in 2004, one case in 2005, one case in 2006, two cases in 2007, one case in 2010, three cases in 2011, three cases in 2012, and one case in 2013 (Fig. 1). There were 20, 20 and 7 subjects from whom type 1, type 2, and type 3 VDPVs were isolated, respectively; both type 2 and 3 VDPVs were isolated from two iVDPV cases (Fig. 2). VDPVs were identified from more than two thirds of provinces in China from 2001–2013 (Fig. 3).

### cVDPVs

A total of 3 cVDPV outbreaks have been reported in China. The first cVDPV outbreak occurred in 2004. From May to July 2004, type 1 VDPVs (with 9–11 nucleotide substitutions in capsid protein VP1 region) were isolated from 2 AFP cases who were diagnosed as clinically compatible polio cases by the provincial PEC and their 3 contacts in Qianxinan Prefecture, Guizhou Province. In addition, another AFP case was also diagnosed as a clinically compatible polio case. Stool specimens collected from the AFP case one month after paralysis onset tested negative for poliovirus, but type 1 VDPV was isolated from stool specimen of his contact. All the 3 AFP cases and 3 of 4 contacts had not been immunized with OPV. The field investigation found that routine immunization had not been delivered in the villages where these AFP cases lived for several years [5]. A convenient coverage survey conducted in the county of the cVDPV outbreak and six adjacent counties found that 23% of children had never received OPV immunization and that the coverage with three doses of OPV ranged from 24% to 67% [5].

From March to May 2006, type 1 VDPV was isolated from one VDPV case and seven healthy contacts in Dahua County of Guangxi Province. Genetic sequencing showed that the isolates diverged with type 1 Sabin strain by 13–20 nucleotides and shared the same 12 nucleotides substitutions within VP1 region, which can be considered to be one single lineage of cVDPV. The VDPV case and all the contacts had not received a dose of OPV. An immunization coverage survey in Dahua County and one adjacent County found that only 61.2% had received three or more doses of OPV among 469 children aged 1 to 10 years. More importantly, the OPV coverage rate was only 27.2% for children aged 5 to 10 years.

Between August 2011 and February 2012, type 2 VDPV (with 6–12 nucleotides substitution in capsid protein VP1 region) were isolated from three AFP cases who were diagnosed as clinically compatible polio cases by the provincial PEC and one contact in Ngawa County, Ngawa Tibetan and Qiang Autonomous Prefecture, Sichuan Province [22]. Additionally, another AFP case from whom type 2 Sabin-related poliovirus strains were isolated with 5 nucleotides VP1 substitution, was diagnosed as clinical compatible polio case by the provincial PEC. Genetic sequencing showed that the isolates diverged with type 2 Sabin strain by 5–12 nucleotides and shared the same 5 nucleotides VP1 substitution, which can be considered to be one single lineage of cVDPV. All four AFP cases lived in Ngawa County and had not been immunized with OPV, whereas the contact had received 3 doses of OPV vaccination.

### iVDPV

A total of four cases were found to be excreting iVDPV (indicating prolonged infections) in China, and all of them were detected after onset of AFP.

Anhui Province. A boy, 2 years of age, with X- linked agammaglobulinemia associated immunodeficiency, who had been immunized with 5 doses of OPV, developed AFP in August 2005 and died in March 2007 from severe pneumonia. Type 2 iVDPV (1.1%–4.2% VP1 divergence) and type 3 iVDPV (2.2%–3.9% VP1 divergence) were isolated from 38 consecutive stool specimens. The duration of virus excretion was about 20 months since the collection of stool specimen with the first virus isolation.

Ningxia Province. A boy aged 2.3 years with congenital agammaglobulinemia who had received 3 doses of OPV, developed AFP in February 2011, 13 months after receipt of the third dose of OPV and died in January 2012. Type 2 iVDPV (2.0%–3.4% VP1 divergence) was isolated from 12 consecutive stool specimens.

Tianjin City. A boy aged 11 months with congenital immunodeficiency disorder who have received 3 doses of OPV, developed AFP in February 2012, 6 months after receipt of the most recent OPV dose. Type 2 iVDPV (1.3%–1.7% VP1 divergence) and type 3 iVDPV (1.3%–2.3% VP1 divergence) were isolated from 12 consecutive stool specimens.

Jiangxi Province. Type 2 iVDPV (6 nucleotides VP1 substitution) and type 3 iVDPV (1.2%–2.1% VP1 divergence) were isolated in 2013 from a boy aged 7 months with congenital immunodeficiency disorder who received 3 doses of OPV and developed AFP in May 2013, 3 months after receipt of the most recent OPV dose.

### Imported VDPVs case

In 2012, a boy aged 18 months, who came from Myanmar, developed AFP in May 2012 and went to a hospital in Yunnan Province for treatment. The child was reported as an AFP case and stool specimens were collected by the local CDC in Yunnan Province. Type 1 VDPV was isolated from both stool specimens with 21 (2.3%) nucleotides VP1 substitution. The child had no previous history of OPV vaccination. It was found that most areas were not covered for routine immunization in the township where the VDPV case lived in Myanmar [23].

### VDPV surveillance among healthy children

VDPV was isolated from 15 contacts of AFP cases and 4 healthy children without direct contact with AFP cases. There were seven contacts in Guangxi Province, four in Guizhou Province and four in other provinces (each one in Sichuan, Shanxi, Fujian and Zhejiang Provinces, respectively). VDPV was isolated from seven contacts of one VDPV case in Guangxi Province in 2006. The five children in Guizhou and Sichuan Province were contacts of the cVDPV case. The four healthy children came from Shanghai (*n* = 2), Guangdong (*n* = 1) and Tibet (*n* = 1).

## Discussion

Many independent VDPV cases were detected in China due to OPV usage in routine immunization, its large population, high population densities, poor sanitation in some areas, and large areas with subtropical and tropical climates [24]. VDPV was isolated from a total of 49 children in China from 2001–2013, including six cVDPV cases (three cVDPV outbreak lineages), four iVDPV cases and five aVDPV cases. Type 1 VDPV represented the main source of VDPV up until 2007, but type 2 VDPVs dominated since 2009 due to the change in the virological definition of type 2 VDPVs. In addition, type 2 VDPV with seven mutations in the VP1 coding region was isolated by environmental surveillance of poliovirus in sewage in December 2012 [25]. The summary of VDPV surveillance is useful for understanding VDPV’s biological properties and establishing vaccination strategies for the final stages of polio eradication.

There were three instances of cVDPV outbreaks in China. Sub-optional population immunity against poliovirus is the most significant risk factor for cVDPV outbreaks, as demonstrated by other studies [11, 15, 26]. In our investigation, all cVDPV cases had not been immunized with OPV. OPV coverage has been reported to be over 99% in China [27], but OPV coverage is generally slightly lower among floating populations in areas with higher economic development and among hard-to-reach populations in remote poor areas [18], such as the mountainous areas in Sichuan, Guangxi and Guizhou Province where the cVDPV outbreaks occurred, as well as some local areas in Xinjiang Uyghur Autonomous Region where an outbreak was confirmed following WPV importation in 2011 [8]. The experience of cVDPV outbreaks and WPV importation in China highlights the risk in polio-free countries with overall high immunization coverage, but immunization gaps existing in high-risk areas. Hence, until all wild, vaccine-related and Sabin polioviruses are eradicated in the world, it is necessary for polio-free countries not only to maintain high quality routine immunization, but also to fill in the immunization gaps in high risk areas by identifying underserved communities and improving immunization capacity in these areas. Regular and comprehensive risk assessment may be a good choice for identifying underserved populations. More efforts should be focused on improving and sustaining access to underserved populations in remote, poor and rural areas in future immunization activities which will increase overall population immunity and reduce the risk of poliovirus transmission.

VDPV with more than 15 nucleotide substitutions in the VP1 region was isolated from five (13.9%) children, and VDPVs with 15 or less nucleotide substitutions were isolated from 31 (86.1%) children after excluding the type 2 VDPVs with <1% divergence. The present genetic characterization of VDPV indicates short-term survival of these strains [18], and that they were not as old as the known cVDPV in other countries [15, 26]. It is assumed that poliovirus genomes evolve at a rate of approximately 1% per year, and >1% nucleotide substitutions is presumed to have replicated for at least 1 year in one or more persons after OPV administration [20]. The sensitive AFP surveillance system in China allows poliovirus to be detected early with a timely emergency response. Maintaining a high quality AFP system plays a key role in identifying and preventing the widespread circulation of cVDPV strains.

A total of four iVDPV cases were found in China from 2001–2013, and the longest duration of virus excretion was about 20 months. Longterm iVDPV excretors present a serious health risk in the post-OPV era. Although there are no reports of secondary cases of iVDPV transmission, whether iVDPV can be transmitted is unknown. It is likely that those who are in close contact have developed sufficient immunity against poliovirus by vaccination. It has been described that immunodeficient patients can prolong excretion of virus for several years. An individual in the United Kingdom excreted VDPV for about 30 years without any paralytic symptoms [28]. An exceptional VAPP case in United States took 6.9 years from initial infection to the onset of paralysis [29]. Immunodeficient persons with chronic infection may represent a potential reservoir for polioviruses in the post-OPV era and creates a major challenge for the end game of the polio eradication initiative. The identification of iVDPV excretors remains a major challenge as the vast majority of long-term iVDPV excretors experience no polio symptoms [30, 31]. Fortunately, poliovirus antiviral agents have been evaluated with the goal to treat iVDPV excreters and to halt excretion in asymptomatic carriers [32].

There was only one event of VDPV case importation in history in China, although WPV importation had been detected many times. Immediately after confirmation of the VDPV case, the China CDC informed WHO and Myanmar about the VDPV case and shared information about the case with them [23]. Based on epidemiological information which was timely provided by China, Myanmar pinpointed the VDPV case and conducted a timely emergency response. The collaboration between China and Myanmar may have prevented the transmission of VDPV, and this effort should be a model example of cross-border collaboration for global polio eradication [23]. The collaboration and coordination must be strengthened in the post-eradication era, such as synchronized cessation of OPV in the world.

## Conclusion

Until all wild, vaccine-related and Sabin polioviruses are eradicated worldwide, high quality routine immunization and sensitive AFP surveillance should be maintained, focusing more efforts on underserved populations in high risk areas. The collaboration and coordination between all countries should be emphasized in post-eradication era for the final success against polio.

## Abbreviations

AFP: Acute flaccid paralysis
aVDPVs: Ambiguous vaccine-derived polioviruses
CDC: Center for Disease Control and Prevention
cVDPVs: Circulating vaccine-derived polioviruses
ELISA: Enzyme-linked immunosorbent assay
GPEI: Global Polio Eradication Initiative
IQR: Interquartile range
ITD: Intratypic differentiation
iVDPVs: Immunodeficiency-associated VDPVs
OPV: Oral attenuated poliomyelitis vaccine
PCR: Polymerase chain reaction
PEC: Provincial Polio Expert Committee
SIAs: Supplementary immunization activities
VAPP: Vaccine-associated paralytic poliomyelitis
VDPVs: Vaccine-derived polioviruses
WHO: World Health Organization
WPV: Wild polioviruses
